# Anti-inflammatory activity of Chios mastic gum is associated with inhibition of TNF-alpha induced oxidative stress

**DOI:** 10.1186/1475-2891-10-64

**Published:** 2011-06-06

**Authors:** Angelike Triantafyllou, Alfiya Bikineyeva, Anna Dikalova, Rafal Nazarewicz, Stamatios Lerakis, Sergey Dikalov

**Affiliations:** 1 Division of Cardiology, Emory University School of Medicine, Atlanta, Georgia, USA; 2 Medical School of Athens, Athens, Greece

**Keywords:** Inflammation, oxidative stress, antioxidant, Chios mastic gum, superoxide, hydrogen peroxide, protein kinase C, NADPH oxidase, TNF-alpha, angiotensin II

## Abstract

**Background:**

Gum of Chios mastic (*Pistacia lentiscus var. chia) *is a natural antimicrobial agent that has found extensive use in pharmaceutical products and as a nutritional supplement. The molecular mechanisms of its anti-inflammatory activity, however, are not clear. In this work, the potential role of antioxidant activity of Chios mastic gum has been evaluated.

**Methods:**

Scavenging of superoxide radical was investigated by electron spin resonance and spin trapping technique using EMPO spin trap in xanthine oxidase system. Superoxide production in endothelial and smooth muscle cells stimulated with TNF-α or angiotensin II and treated with vehicle (DMSO) or mastic gum (0.1-10 μg/ml) was measured by DHE and HPLC. Cellular H_2_O_2 _was measured by Amplex Red. Inhibition of protein kinase C (PKC) with mastic gum was determined by the decrease of purified PKC activity, by inhibition of PKC activity in cellular homogenate and by attenuation of superoxide production in cells treated with PKC activator phorbol 12-myristate 13-acetate (PMA).

**Results:**

Spin trapping study did not show significant scavenging of superoxide by mastic gum itself. However, mastic gum inhibited cellular production of superoxide and H_2_O_2 _in dose dependent manner in TNF-α treated rat aortic smooth muscle cells but did not affect unstimulated cells. TNF-α significantly increased the cellular superoxide production by NADPH oxidase, while mastic gum completely abolished this stimulation. Mastic gum inhibited the activity of purified PKC, decreased PKC activity in cell homogenate, and attenuated superoxide production in cells stimulated with PKC activator PMA and PKC-dependent angiotensin II in endothelial cells.

**Conclusion:**

We suggest that mastic gum inhibits PKC which attenuates production of superoxide and H_2_O_2 _by NADPH oxidases. This antioxidant property may have direct implication to the anti-inflammatory activity of the Chios mastic gum.

## Introduction

*Chios Mastic gum *is derived from *Pistacia lentiscus *var. *Chia *cv. Anacardiaceae, a plant which is mainly met on the greek island of Chios. The beneficial, healing properties of mastic gum have been known since antiquity [1]. From Dioscurides and Galenus to the 'Jerusalem Balsam', *Pistacia lentiscus *has been traditionally considered as a medical agent and is incorporated in Mediterranean cuisine, as a therapeutic means for hepatic inflammation, for disorders of the stomach and intestine, and beneficial for the teeth [2]. Nowadays, food products (apart from the well-established chewing gum) and cosmetics based on mastic have been created. Chios mastic gum is consumed as chewing gum and also in other culinary art usage, especially in Greek, Turkish and Arabic kitchens, i.e. in the powder form as food additive, in the form of sugar containing gel as a sweetener and as mastic oil as a sweet additive in drinks. It has been referred to over centuries as having medicinal properties to treat a variety of diseases. It has been proven as a therapeutic agent against various gastric malfunctions, such as gastralgia, dyspepsia and gastric ulcer [3,4].

Clinical studies have emphasized anti-inflammatory activity of Chios mastic gum [5,6]. This biological activity can be attributed to a variety of compounds. It contains triterpenes of the oleanane, euphane and lupine type [7,8]; alpha-tocopherol and polyphenols; the latter have been associated with a hypotensive effect of mastic [9]. *Chios mastic *possesses anti-bacterial activity [10,11], for which verbenone, alpha-terpineol, and linalool seem to be responsible.

*Pistacia lentiscus *has been traditionally regarded also as an anti-cancer agent, especially on tumours of breast, liver, stomach, spleen, and uterus. Surprisingly enough, these traditional beliefs are in line with recent studies demonstrating that *Chios mastic *induces apoptosis and possesses antiproliferative activity in colon cancer cells [12]. *Pistacia lentiscus *has already been associated with cardiovascular protection and hepatoprotection [13]. It inhibits human LDL oxidation and acts on peripheral blood mononuclear cells to elicit an antiatherogenic effect [14]. The aqueous extract from the leaves of *Pistacia lentiscus *demonstrated hepatoprotective effect in rats intoxicated with carbon tetrachloride [15], which is well-known for induction of oxidative stress. The antiatherogenic activity and protection from carbon tetrachloride toxicity are likely to be associated with antioxidant properties of mastic gum. However, free radical scavenging properties and antioxidant activity of mastic gum has not been investigated.

Inflammation is strongly associated with oxidative stress induced by TNF-α and angiotensin II [16]. Pro-inflammatory cytokine TNF-α stimulates of superoxide production by NADPH oxidases [17] which provides feed-forward activation of inflammatory pathways [18]. We have hypothesized that anti-inflammatory activity of Chios mastic gum is associated with its potential antioxidant activity. In this work, we have evaluated the free radical scavenging and antioxidant activity of Chios mastic gum on TNF-α and angiotensin II - induced superoxide production. We found that mastic gum inhibited superoxide production induced by both TNF-α and angiotensin II, which may have direct implications for its physiological activity.

## Materials and methods

### Reagents

Mastic gum was obtained from Chios Mastiha Growers Association (Chios, Greece) and dissolved in DMSO. Typical mastic gum contained 40-55% triterpenic acids and 20-25% polymer fraction (poly-β-myrcene). Angiotensin II (Ang II), superoxide dismutase (SOD), TNF-α, 12-myristate 13-acetate (PMA) and xanthine were obtained from Sigma-Aldrich (St. Louis, MO). Xanthine oxidase was purchased from Roche Molecular Biochemicals (Indianapolis, IN). 1-Hydroxy-3-carboxy-2,2,5,5-tetramethyl-pyrrolidine (CPH) and 5-ethoxycarbonyl-5-methyl-1-pyrroline N-oxide (EMPO) were purchased from Enzo Life Sciences (Plymouth Meeting, PA). Dihydroethidium was purchased from Molecular Probes (Eugene, OR).

### Electron Spin Resonance (ESR) experiments

ESR experiments were carried out in 50 mM sodium phosphate buffer (pH 7.4) with 0.9% NaCl and DTPA (100 μM). ESR spectra were recorded at room temperature using an EMX ESR spectrometer (Bruker BioSpin, Massachusetts). EMPO samples ESR spectra were recorded with the following settings: field sweep, 70 G; microwave frequency, 9.78 GHz; microwave power, 40 mW; modulation amplitude, 0.7 G; conversion time, 40.96 ms; time constant, 40.96 ms. Superoxide detection by CPH was performed by following the low-field peak of the nitroxide ESR spectra using time scans with the following settings: microwave frequency, 9.78 GHz; modulation amplitude, 2 G; microwave power, 20 mW; conversion time, 1.3 s; time constant, 5.2 s.

### Superoxide radical generation

The xanthine oxidase superoxide generating system contained xanthine oxidase (20 mU/ml), xanthine (50 μM), and DTPA (0.1 mM) in 50 mM sodium phosphate buffer (pH 7.4) with 0.9% NaCl.

### Simulation of ESR spectra

Computer simulation of experimental ESR spectra was used for the calculation of hyperfine coupling constants. Programs for the simulation of ESR spectra and the spin-trap database are readily available to the public through the Internet http://epr.niehs.nih.gov/[19].

### Cell culture

Rat aortic smooth muscle cells (RASMC) were isolated from rat aortas by enzymatic digestion as previously described [20]. Quiescent cells were prepared in Dulbecco's modified Eagle's medium (DMEM) containing 10% calf serum, 4.5 g/l glucose and 2 mM glutamine by placing 70% confluent RASMC in serum-free media for three days. Bovine aortic endothelial cells (BAEC, passage 4 to 8) were cultured in Media 199 containing 10% fetal calf serum supplemented with 2 mM L-glutamine and 1% vitamins. On the day before the study, the fetal calf serum concentration was reduced to 1%. Confluent cells were used for the experiments.

All animal use complied with National Institutes of Health guidelines and was approved by the Emory University Institutional Animal Care and Use Committee.

### Detection of intracellular superoxide with high-performance liquid chromatography

To evaluate intracellular production of superoxide, we measured the formation of 2-hydroxyethidium from dihydroethidium (DHE) using high-performance liquid chromatography (HPLC) analysis as recently reported [21]. Medium was removed from cells platted on 100 mm dish and replaced with 10 μM DHE in fresh Krebs-HEPES buffer. After 20 minute incubation at 37°C buffer was aspirated, cells were collected into 300 μL methanol, and cell homogenate was filtered via 0.2 μm filter. Separation of ethidium, 2-hydroxyethidium, and dihydroethidium was performed with the use of an acetonitrile gradient and a C-18 reverse-phase column (Nucleosil 250-4.5 mm) on a Beckman HPLC system with a fluorescence detector Jasco FP-2020 (Easton, MD) using an emission wavelength of 580 nm and an excitation of 480 nm.

### Measurements of NADPH oxidase activity in membrane fraction

The activity of NADPH oxidase was directly measured by NADPH dependent O_2_* production in the membrane fractions of RASMC prepared as described previously [22].

### Measurements of cellular H_2_O_2_

Production of cellular H_2_O_2 _was analyzed by Amplex Red assay provided by Invitrogen (Carlsbad, CA) based on conversion of Amplex Red (50 μM) into fluorescent resorufin in the presence of horseradish peroxidase (0.1 units/ml) after two hours incubation with cells [21].

### The PKC Kinase Activity Assay

The PKC Kinase Activity Assay was obtained from Enzo Life Sciences (Plymouth Meeting, PA). This is based on a solid phase enzyme-linked immuno-absorbent assay (ELISA) utilizing a specific synthetic peptide as a substrate for PKC and a polyclonal antibody recognizing the phosphorylated form of the substrate phosphorylated by PKC. ATP is added to initiate the reaction, and kinase reaction is terminated by Phosphospecific Substrate Antibody to bind specifically to the phosphorylated peptide substrate. The phosphospecific antibody is subsequently bounded by a peroxidase conjugated secondary antibody. The assay is developed with tetramethylbenzidine substrate and a color develops in proportion to PKC phosphotransferase activity. The intensity of the color is measured in microplate reader at 450 nm.

### Statistical analysis

Data are presented as mean ± standard error. For comparison of two groups, a two-tailed *t*-test was employed using Excel software. Statistical significance was assumed when *p *< 0.05.

## Results

### Inhibition of TNF-α induced superoxide production

**Figure 1:**
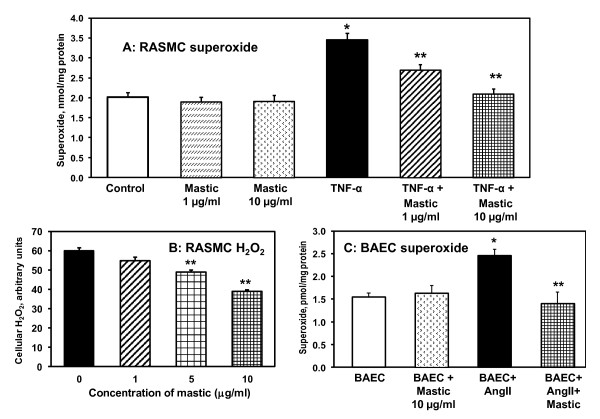
Effect of mastic gum on cellular production of O_2_* and H_2_O_2_. (A) Production of intracellular O_2_* was measured by DHE following accumulation of 2-hydroxyethidium using HPLC as described in Materials and Methods [21]. RASMC were stimulated with 20 ng/ml TNF-α for 4-hours. Cells were supplemented with various doses of mastic gum (0-10 μg/ml) for 15-minutes prior to measurements of superoxide. (B) Production of cellular H_2_O_2 _was measured by Amplex Red as described in Materials and Methods [21]. RASMC were stimulated with 20 ng/ml TNF-α for 4-hours and then supplemented with various doses of mastic gum (0-10 μg/ml) for 15-minutes prior to measurements of H_2_O_2_. *P < 0.01 vs Control, **P < 0.01 vs TNF-α. (C) Superoxide production in bovine aortic endothelial cells (BAEC) stimulated with 200 nM angiotensin II (Ang II) for 4-hours and treated with mastic gum. Control unstimulated BAEC or Ang II-stimulated BAEC were supplemented with 10 μg/ml mastic gum for 15-minutes prior to measurements of superoxide. Data are average from six to eight separate experiments ± Standard Error. *P < 0.01 vs Control, **P < 0.01 vs Ang II.

It is well documented that TNF-α induced superoxide production by NADPH oxidases plays an important role in inflammatory injury [23]. We have investigated the effect of mastic gum on TNF-α induced superoxide production in cultured RASMC. It has been found that 4-hour treatment of RASMC with TNF-α lead to 2-fold increase in cellular superoxide (Figure 1A) measured by accumulation of superoxide specific product of dihydroethidium [21]. Supplementation of TNF-α stimulated cells with mastic gum lead to dose-dependent decrease in cellular superoxide but did not affect superoxide production in unstimulated cells. Interestingly, mastic gum inhibited not only TNF-α stimulated superoxode production but reduced cellular H_2_O_2 _(Figure 1B).

These data can be explained by three possible mechanisms: 1) mastic gum scavenges the superoxide resulting in lower amount of detectable superoxide; 2) mastic gum act as superoxide dismutase mimetic which decreases superoxide detection but does not affect the amount H_2_O_2_; 3) mastic gum inhibits superoxide production by affecting cellular sources of superoxide such as NADPH oxidases. We have investigated all three mechanisms.

### Investigation of superoxide scavenging by mastic gum

Xanthine oxidase and xanthine are commonly used as a superoxide-generating system [24]. Superoxide produced by this enzyme can be detected by spin traps such as EMPO, which produces stable EMPO/*OOH radical adduct [25]. We have investigated the potential scavenging of superoxide by mastic gum by electron spin resonance (ESR) with spin trap EMPO.

**Figure 2:**
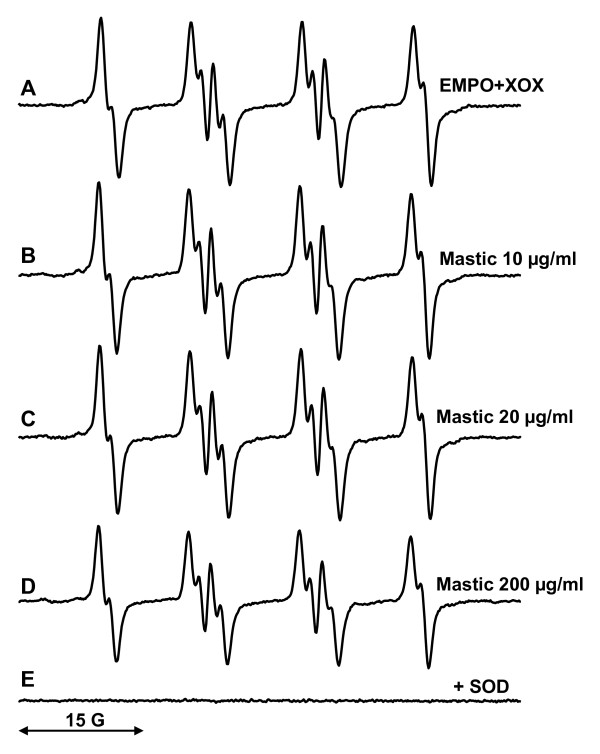
**Spin trapping study of superoxide scavenging by mastic gum**. (A) ESR spectrum of EMPO (60 mM) with xanthine (50 μM) and xanthine oxidase (20 mU/ml); (B) ESR spectrum of (A) plus 10 μg/ml mastic gum; (C) ESR spectrum of (A) plus 20 μg/ml mastic gum; (D) ESR spectrum of (A) plus 200 μg/ml mastic gum; (E) ESR spectrum of (A) plus 50 U/ml Cu, Zn-SOD. Computer simulation of ESR spectra (hyperfine coupling constants a^N ^= 13.3 G, a^H^_β _= 10.8 G, a^H^_γ _= 1.1 G) [41] and inhibition by SOD (E) confirmed detection of EMPO/*OOH radical adduct. ESR settings were as described in Materials and Methods.

ESR spectrum of sample containing xanthine oxidase, xanthine and EMPO revealed typical spectrum of EMPO/*OOH radical adduct (Figure 2A). Addition of 10 μg/ml and 20 μg/ml mastic gum to xanthine oxidase superoxide-generating system did not significantly affect the formation of EMPO/*OOH radical adduct (Figure 2B, C). Supplementation of xanthine oxidase superoxide-generating system with 200 μg/ml mastic gum lead only to small 16% decrease in EMPO/*OOH ESR signal (Figure 2E). Meanwhile, supplementation of xanthine oxidase superoxide-generating system with well-known superoxide scavenger ascorbate (5 μg/ml) [26] or 100 U/ml superoxide dismutase (Figure 2E) completely abolished the formation of EMPO/*OOH. These data shows that mastic gum does not have significant superoxide scavenging activity.

It is possible that mastic gum does not scavenge superoxide but stimulates its intracellular dismutation or interfere with its reaction with dihydroethidium. In these cases mastic gum should not change the production of cellular H_2_O_2_. In order to test this hypothesis we analyzed the production of cellular H_2_O_2 _in TNF-α treated cells by Amplex Red [21]. It was found that mastic gum inhibited the production of cellular H_2_O_2 _in dose dependent manner (Figure 1B). These data argue that mastic gum did not scavenge or dismutate superoxide but actually inhibited the production of cellular reactive oxygen species.

### Inhibition of TNF-α stimulated NADPH oxidase

NADPH oxidases are one of the main sources of cellular superoxide [27,28]. We have analyzed possible inhibition of NADPH oxidases by mastic gum. Activity of NADPH oxidases was determined by NADPH-depended superoxide production in the membrane fractions of RASMC before and after cell stimulation with TNF-α measured by ESR [22].

**Figure 3:**
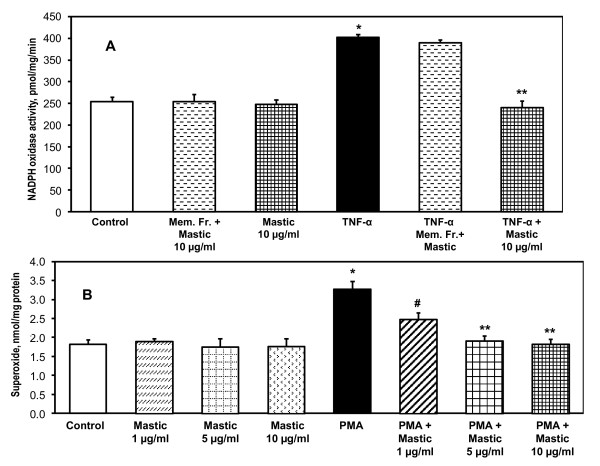
**Inhibition of NADPH oxidase in TNF-α stimulated cells treated with mastic gum and attenuation PMA-stimulated superoxide production**. (A) Activity of NADPH oxidase was measured as NADPH-dependent O_2_* production in membrane fractions using ESR as described in Materials and Methods [22]. NADPH oxidase activity was analyzed in membrane fractions of control unstimulated RASMC or RASMC stimulated with 20 ng/ml TNF-α for 4-hours. Mastic gum (10 μg/ml) was applied for 15-minutes prior to isolation of membrane fractions. Direct supplementation of mustic gum to membrane fractions isolated from control or TNF-α stimulated RASMC did not affect NADPH oxidase activity. Data are average from three to six separate experiments ± Standard Error (*P < 0.01 vs Control, **P < 0.01 vs TNF-α). (B) Production of intracellular O_2_* was measured by DHE following accumulation of 2-hydroxyethidium using HPLC [21] in control or PMA-stimulated RASMC (1 μM PMA, 4-hours) supplemented with various doses of mastic gum (0-10 μg/ml) for 15-minutes prior to measurements of superoxide. Data are average from six separate experiments ± Standard Error (*P < 0.01 vs Control, ^#^P < 0.05 vs TNF-α, **P < 0.01 vs TNF-α).

It has been found that supplementation of the isolated membrane fraction with mastic gum did not affect the basal NADPH oxidase activity (Figure 3A). Treatment of unstimulated RASMC with mastic gum did not change the NADPH oxidase activity. Stimulation of RASMC with TNF-α significantly increased NADPH oxidase activity. Supplementation of the isolated membrane fraction with mastic gum did not affect the TNF-α induced NADPH oxidase activity. Interestingly, treatment of TNF-α stimulated RASMC with mastic gum completely abolished an activation of NADPH oxidase (Figure 3A). These data show that mastic gum did not directly inhibited NADPH oxidase. Mastic gum inhibited TNF-α stimulated NADPH oxidases only in intact cells and it did not affect the basal activity. This may suggest that mastic gum may attenuate activation of NADPH oxidases such as PKC-mediated phosphorylation of p47phox subunit of NADPH oxidase [29]. We, therefore, investigated the effect of mastic gum on stimulation of NADPH oxidase by PKC activator 12-myristate 13-acetate (PMA) [29,30].

### Inhibition of PMA-stimulated NADPH oxidase

Treatment of cells with PMA provides specific PKC-dependent activation of NADPH oxidases [30]. We analyzed the effect of mastic gum on PMA-stimulated superoxide production (Figure 3B). It was found that mastic gum inhibited PMA-stimulated superoxide production in RASMC in dose dependent manner (Figure 3B). Similar to TNF-α induced superoxide production, 10 μg/ml mastic gum completely abolished the PMA-stimulated superoxide production. These data showed that mastic gum inhibited PKC which attenuates activation of NADPH oxidase. We suggest that PKC inhibition by mastic gum is responsible for attenuation of TNF-α induced oxidative stress.

### The PKC Kinase Activity Assay

**Figure 4:**
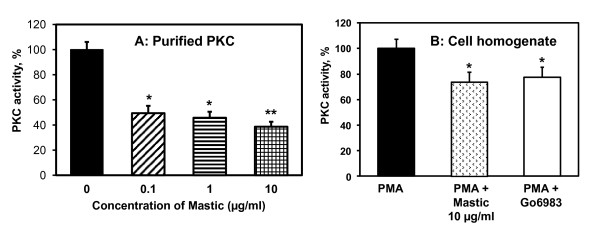
**Inhibition of PKC activity by mastic gum**. PKC kinase activity was measured by ELISA-based assay from Enzo Life Sciences in the samples with purified PKC (60 ng) or homogenate of PMA-stimulated RASMC (30 μg). Purified PKC was briefly incubated with 0, 0.1, 1.0 or 10 μg/ml mastic gum prior to PKC activity assay (A). PMA-stimulated RASMC were supplemented with 10 μg/ml mastic gum, 1 μM Go6983 or DMSO as a vehicle for 15-minutes prior to measurements of PKC activity. Data are average from four separate experiments ± Standard Error (*P < 0.01 vs Control, **P < 0.05 vs 0.1 μg/ml Mastic gum).

To determine if mastic gum indeed directly inhibits PKC we have measured purified PKC activity and PKC activity in cellular homogenate using ELISA-based PKC Kinase Activity Assay from Enzo Life Sciences in the presence of various concentrations of mastic gum. It was found that activity of purified PKC was inhibited by mastic gum in the dose dependent manner down to 60% (Figure 4A). PKC activity in homogenate of PMA-stimulated RASMC was significantly inhibited by 10 μg/ml mastic gum. Interestingly, supplementation of potent PKC-α and PKC-β inhibitor Go6983 to PMA-stimulated RASMC inhibited total PKC activity to similar level as in mastic gum supplemented RASMC (Figure 4B). These data support the direct inhibition of PKC by mastic gum.

### Inhibition of angiotensin II stimulated superoxide production

PKC-dependent activation of NADPH oxidases plays an important role in various pathological conditions associated with oxidative stress. Angiotensin II is one of the key agonists, which stimulates vascular NADPH oxidases via PKC-dependent pathway. In this work we have investigated the effect of mastic gum on angiotensin II induced superoxide production in bovine aortic endothelial cells (Figure 1C). It was found that 4-hour stimulation of BAEC with angiotensin II significantly increased cellular superoxide production. Interestingly, 15-minute treatment with mastic gum after angiotensin II stimulation completely abolished angiotensin II induced oxidative stress (Figure 1C). These data support that mastic gum inhibits the production of cellular superoxide by PKC inhibition.

## Discussion

**Figure 5:**
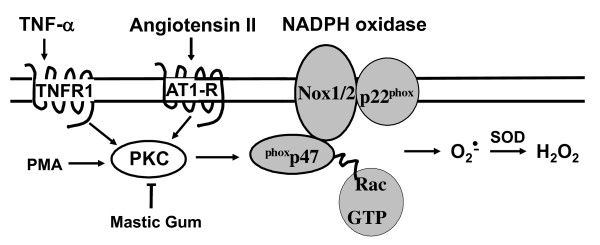
**Proposed mechanism of antioxidant activity of mastic gum via inhibition of PKC-dependent activation of NADPH oxidases**.

In this work for the first time we report that mastic gum block TNF-α induced superoxide production. The decrease in superoxide production was associated with inhibition of NADPH oxidase in TNF-α stimulated smooth muscle cells. It has been found that inhibition of PKC is likely to be responsible for the down regulation of NADPH oxidase activity in mastic gum treated cells. Mastic gum also attenuated superoxide production in angiotensin II - stimulated endothelial cells, which strongly support the general antioxidant activity of mastic gum via inhibition of PKC (Figure 5).

There are different kinds of antioxidants. Many antioxidants are free radical scavengers which react directly with reactive oxygen species such as ascorbate, vitamin E, superoxide dismutase and catalase [31]. Other inhibit the sources of reactive oxygen species, for example, xanthine oxidase inhibitor allopurinol, NADPH oxidase inhibitor apocynin, angiotensin II receptor blockers which attenuate activation of NADPH oxidases [32]. These compounds inhibit production of reactive oxygen species directly or indirectly. Direct inhibition occurs due to bind to the source of reactive oxygen species, while affecting the regulation of these enzymes may provide indirect inhibition. We suggest that mastic gum inhibits one of the main cellular sources of superoxide and H_2_O_2 _indirectly by blocking the PKC-dependent activation of NADPH oxidases (Figure 5).

Protein kinase C plays diverse roles in many cellular functions, notably proliferation, differentiation, and cell survival [33]. Members of the PKC family are key signaling mediators in immune responses, and pharmacological inhibition of PKC may be useful for treating immune-mediated diseases [34]. PKC-dependent activation of NADPH oxidases is an important step in development of oxidative stress in cardiovascular diseases, diabetes and inflammation [35,36]. However, various ATP-competitive inhibitors of PKC have problems with specificity [33]. Interestingly, inflammation induced by periodontitis has been link to cardiovascular diseases and it has been associated with PKC-mediated enhanced oxidative stress [36]. Meanwhile, mastic gum is commonly used for treatment of gum diseases such as periodontitis [37], which may partially explain beneficial effect of mastic gum use.

Mastic gum has been reported to inhibit cell proliferation and block the cell cycle progression which may be important in anticancer activity of mastic gum [38]. However, this effect was observed at concentrations of mastic gum higher that 20 μg/ml, while our data showed that 1 μg/ml of mastic gum significantly inhibited PKC activity (Figure 4A) and attenuated PMA-stimulated superoxide production (Figure 3B). These data suggest that mastic gum may affect multiple cellular targets via PKC dependent pathways.

The chemical structures of mastic gum components responsible for these activities are not clear. It has been previously reported that ten triterpenes were identified in the acidic fraction of mastic gum and 26 triterpenes were characterized in the neutral fraction [7,8]. It is interestingly to speculate that inhibition of PKC and NF-kappaB pathways is associated with triterpene content rather than minor presence of tocopherol and polyphenols because of the lack of significant free radical scavenging by mastic gum. However, further studies are required in order to elucidate specific bioactive components of mastic gum.

## Conclusion

Chios mastic gum has been extensively used for centuries in Mediterranean and Middle Eastern countries, both as a nutritional supplement and herbal remedy. Medical trials show that gum mastic may have cytoprotective or anti-acid effects on the gastrointestinal system [4]. Recent studies seem to suggest that gum mastic may exhibit antibacterial properties [39] and inhibit the proliferation of androgen-dependent prostate cancer [40]. This work makes an important addition to this list demonstrating potential role of antioxidant properties in the anti-inflammatory activity of the Chios mastic gum based on inhibition of PKC-dependent NADPH oxidases.

## Competing interests

The authors declare that they have no competing interests.

## Authors' contributions

The authors' contributions were as follows: AT and SD contributed to the design of the study and drafted the manuscript; AB carried out the measurements of cellular superoxide by HPLC. AD performed analysis of NADPH oxidase activity and cellular H_2_O_2_. RN participated in the study of PKC activity. SL performed the statistical analysis and helped to draft the manuscript. SD made critical revisions of the manuscript. All authors read and approved the final manuscript. None of authors had any personal or financial conflict of interest.
